# Perilunate Dislocation

**Published:** 2015-01-30

**Authors:** Tom Reisler, Paul J. Therattil, Edward S. Lee

**Affiliations:** Division of Plastic and Reconstructive Surgery, Department of Surgery, New Jersey Medical School, Rutgers University, Newark, NJ

**Keywords:** perilunate dislocation, Mayflield's classification, scapholunate advanced collapse wrist, Gilula's lines, Terry-Thomas sign

## DESCRIPTION

**Figure 1:**
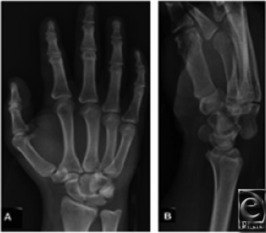
Radiograph demonstration of a perilunate dislocation: The lunate maintains its normal articulation with the radius, whereas the capitated articular surface is dislocated from the lunate dorsally.

A 30-year-old man is brought to the emergency department after a motor vehicle collision. He complains of right wrist pain. Trauma screening shows no other injuries, and wrist radiographs are obtained (Figs 1a and 1b). The right hand is moderately swollen with diffuse tenderness. The findings of wrist and hand neurovascular examinations are normal. Range of motion is limited by pain.

## QUESTIONS

1. **What do the radiographs show?**
2. **What is the mechanism for perilunate dislocation?**
3. **Which ligaments must be ruptured for perilunate dislocation to occur?**
4. **An operating room is not immediately available. What is the most appropriate next step in management?**

## DISCUSSION

**Figure 2:**
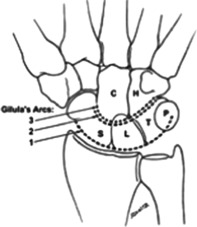
Diagrammatic representation of the 3 carpal arcs of Gilula's lines. The first arc is a smooth curve outlining the proximal convexities of the scaphoid, lunate, and triquetrum. The second arc traces the distal concave surfaces of the same bones, and the third arc follows the main proximal curvatures of the capitate and hamate. Copyright by Pinterest. https://www.pinterest.com/clifdo/ot-anatomy-and-physiology/.

**Figure 3:**
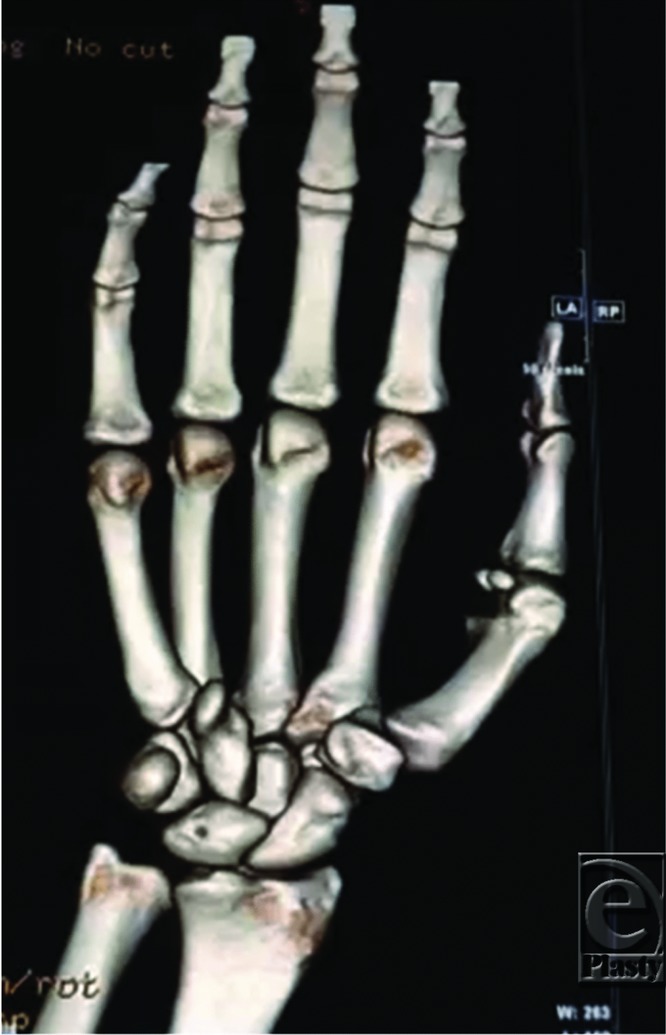
Right wrist 3-dimentional computed tomographic scan demonstration of dorsal perilunate dislocation, and clear evidence of dorsal dislocation of the fourth carpormetacarpal (CMC) joint and subluxation at the fifth CMC joint, as well as dislocation of the scaphoid/trapezium articulation and subluxation at the trapezium/trapezoid articulation.

The initial routine radiographic examination in a patient with a suspected carpal injury should include at least 4 views of the wrist: anteroposterior (AP), lateral, scaphoid, and 45-degree semipronated oblique. In the AP view, 3 fairly smooth radiographic arcs (Gilula's lines) can be drawn to define normal carpal relationships (Fig 2). In perilunate dislocation, as in this case, there is a break in Gilula's arc, the lunate and capitate are overlapped, and the lunate appears triangular “piece-of-pie sign.” On a lateral view, there is loss of colinearity of radius, lunate, and capitate; and the scapholunate (SL) angle is greater than 70°. Articulating bones normally have parallel apposing surfaces separated by 2 mm or less. Any overlap between well-profiled cortices of carpal bones or joint spacing that substantially exceeds that found in the uninjured wrist strongly suggests an intercarpal abnormality. In addition, once a perilunate dislocation is reduced, there is usually a radiographic evidence of scapholunate dissociation as a result of ligamentous injury and flexion of the scaphoid. Anteroposterior radiographs will show an increased scapholunate gap known as the “Terry-Thomas sign”; and a scaphoid “Ring sign” as a result of the distal pole of the scaphoid moving relatively closer to the proximal scaphoid cortex and being viewed end-on.^1^ In the wrist, computed tomographic scans are usually taken at 2-mm intervals. Computed tomography has the added advantage of allowing computer manipulation to obtain 3-dimensional images of the carpal bones, which help visualize the structure to be analyzed, and provides excellent visual information about the amount and direction of any displacement (Fig 3). Interestingly, the earlier patient's radiographs also have evidence of dorsal dislocation of the fourth carpometacarpal (CMC) joint and subluxation at the fifth CMC joint, as well as dislocation of the scaphoid/trapezium articulation and subluxation at the trapezium/trapezoid articulation.

Perilunate dislocations are relatively uncommon injuries; however, they are the most common form of carpal dislocation. Most dorsal perilunate dislocations are the result of an indirect mechanism of injury, usually an extreme extension of the wrist, associated with a variable degree of ulnar deviation and midcarpal supination, often secondary to violent trauma such as sustained from a fall from a height or a motorcycle accident. In patients with a perilunate dislocation, a careful assessment of the neurovascular status is imperative, with particular attention to the median and ulnar nerves, which may be injured by direct contusion at the moment of impact or by compression from displaced bones or swelling in the carpal canal. Associated soft tissue, bone, and joint injuries known to be caused by a similar mechanism (CMC dislocation, radioulnar joint dislocation, radial head fracture, and elbow dislocation) should be specifically sought as well.

Perilunate dislocations can be classified as either greater arc or lesser arc injuries depending on the site of trauma and the extent of carpal bone injury. The pattern of injury traverses both the greater and lesser carpal arcs. Lesser arc dislocations, as in the scenario described earlier, involves injury to the scapholunate, lunocapitate, and/or lunotriquetral ligaments; these injuries are rated as type I to type IV according to Mayfield's classification. Our patient's dorsal perilunate dislocation is classified as Mayfield's stage I to III.^2^^,^^3^ Injury to the scapholunate interosseous ligament and radiolunate ligament, as a result of perilunate dislocation, will result in scapholunate dissociation. Early recognition and treatment are important to prevent the development of radiocarpal arthritis. Surgical repair or reconstruction of the scapholunate interosseous ligament is normally required to prevent long-term complications of a scapholunate advanced collapse wrist.^4^

In the scenario described, with no immediate operating room availability, all perilunate dislocations need to be reduced by closed means in the emergency department under conscious sedation as soon as possible. The reason for such urgency is to decompress the median nerve at the carpal tunnel and to release tension on the vascular supply to the displaced carpal bones.

**Figure 4:**
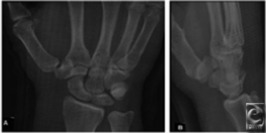
A successful postreduction radiographs demonstrating a smooth Gilula's arc.

An initial period of 10 minutes of uninterrupted finger traps traction with the elbow flexed 90° is helpful before reduction. When the wrist has been distracted for 10 minutes, traction is released, and the method of reduction of dorsal perilunate dislocations described by Tavernier is attempted as follows: with one hand, the patient's wrist is extended (maintaining longitudinal traction), while the thumb of the other hand stabilizes the lunate on the palmar aspect of the wrist. Gradual flexion of the wrist allows the capitate to snap back into the concavity of the lunate. To facilitate this maneuver, the operator's thumb stabilizes the lunate to prevent its being displaced forward by the capitate. When the lunocapitate joint is reduced, and without releasing traction, the wrist is extended gradually while the lunate is pushed dorsally with the thumb, and a full reduction is usually achieved. At this point, traction is released, and the wrist is brought back to neutral. The sooner after the injury this technique is performed, the easier is the reduction. The wrist is initially immobilized with a dorsal short arm and thumb spica plaster splint with the wrist in neutral. Postreduction radiographs must be taken (Fig 4).

The forearm is maintained elevated until a definitive surgical treatment can be instituted. If the dislocation is reduced, delaying the definitive treatment several hours or even a few days to get the right equipment and trained personnel is not a problem, as long as there is no neurovascular compromise.^5^
